# Evaluation of Chemical Fluorescent Dyes as a Protein Conjugation Partner for Live Cell Imaging

**DOI:** 10.1371/journal.pone.0106271

**Published:** 2014-09-03

**Authors:** Yoko Hayashi-Takanaka, Timothy J. Stasevich, Hitoshi Kurumizaka, Naohito Nozaki, Hiroshi Kimura

**Affiliations:** 1 Graduate School of Frontier Biosciences, Osaka University, Suita, Japan; 2 Graduate School of Bioscience and Biotechnology, Tokyo Institute of Technology, Yokohama, Japan; 3 Department of Biochemistry and Molecular Biology, Colorado State University, Fort Collins, Colorado, United States of America; 4 Janelia Farm Research Campus, Howard Hughes Medical Institute, Ashburn, Virginia, United States of America; 5 Graduate School of Advanced Science and Engineering, Waseda University, Tokyo, Japan; 6 Mab Institute Inc, Sapporo, Japan; St. Georges University of London, United Kingdom

## Abstract

To optimize live cell fluorescence imaging, the choice of fluorescent substrate is a critical factor. Although genetically encoded fluorescent proteins have been used widely, chemical fluorescent dyes are still useful when conjugated to proteins or ligands. However, little information is available for the suitability of different fluorescent dyes for live imaging. We here systematically analyzed the property of a number of commercial fluorescent dyes when conjugated with antigen-binding (Fab) fragments directed against specific histone modifications, in particular, phosphorylated H3S28 (H3S28ph) and acetylated H3K9 (H3K9ac). These Fab fragments were conjugated with a fluorescent dye and loaded into living HeLa cells. H3S28ph-specific Fab fragments were expected to be enriched in condensed chromosomes, as H3S28 is phosphorylated during mitosis. However, the degree of Fab fragment enrichment on mitotic chromosomes varied depending on the conjugated dye. In general, green fluorescent dyes showed higher enrichment, compared to red and far-red fluorescent dyes, even when dye∶protein conjugation ratios were similar. These differences are partly explained by an altered affinity of Fab fragment after dye-conjugation; some dyes have less effect on the affinity, while others can affect it more. Moreover, red and far-red fluorescent dyes tended to form aggregates in the cytoplasm. Similar results were observed when H3K9ac-specific Fab fragments were used, suggesting that the properties of each dye affect different Fab fragments similarly. According to our analysis, conjugation with green fluorescent dyes, like Alexa Fluor 488 and Dylight 488, has the least effect on Fab affinity and is the best for live cell imaging, although these dyes are less photostable than red fluorescent dyes. When multicolor imaging is required, we recommend the following dye combinations for optimal results: Alexa Fluor 488 (green), Cy3 (red), and Cy5 or CF640 (far-red).

## Introduction

Live cell fluorescence imaging has been a powerful and common technique in cell biology. To acquire high quality and high resolution images without damaging cells, it is essential to optimize the imaging conditions by choosing appropriate objective lenses, filter sets, and detectors for the microscope. In addition, the choice of an appropriate fluorophore is also an important factor. To analyze protein localization in living cells, tagging with a fluorescent protein has become popular, as genetically encoded systems are convenient and many different fluorescent proteins are now available with a range of properties [1], [2], [3]. Nevertheless, small chemical fluorescent dyes can still be useful for labeling and tracking specific proteins in living cells [4], [5], [6], [7], [8]. Genetically encoded peptide-tags, such as HaloTag, SNAP, CLIP and tetracystein, can be labeled with fluorescent dyes conjugated with their specific ligands. These tags are particularly useful for distinguishing preexisting from newly synthesized protein molecules by pulse labeling [9] and for single molecule tracking since chemical dyes such are generally more photostable than fluorescent proteins [8]. Some fluorescent dyes have also allowed super-resolution imaging in living cells [10]. Besides genetically encoded systems, the behavior of proteins can also be directly tracked by injecting dye-conjugated protein molecules into cells. For example, nuclear division cycles in early *Drosophila* embryos were visualized using fluorescent dye-conjugated histone [11]. Intracellular endogenous protein or posttranslational modifications have also been monitored using fluorescently labeled antigen binding (Fab) fragments, which were prepared from the specific antibodies [12], [13], [14], [15], [16]. Fab-based live endogenous modification labeling (FabLEM) has been particularly useful for tracking histone modifications in living cells [14], [15].

In principle, any fluorescent dye can be chosen as a protein conjugation partner for live cell imaging, and many new dyes with improved photochemical properties have been developed [17], [18]. However, in addition to photochemical properties, like brightness and photostability, it is important for live cell applications to assess the effects of dye-conjugation on protein function as well as the propensity of dye binding to cellular components. Indeed, some dyes are known to target specific organelles like mitochondria [19], [20]. We have also noticed, during the course of FabLEM experiments, that the cytoplasmic background signals appear different when using the same Fab labeled with different dyes [15]. A recent single molecule analysis has shown that the property of dyes affect the binding of conjugated protein that binds to epidermal growth factor receptor on plasma membrane (anti-EGFR affibody) [21].

Here we systematically evaluated the suitability of a number of commercially available dyes with different excitation wavelengths (at around 488, 550, and 640 nm, for green, red, and far-red dyes, respectively) for intracellular fluorescence live imaging.

## Materials and Methods

### Ethics statement

All animal care and experimental procedures in this study were approved by Hokkaido University Animal Experiment Committee (approval number: 11-0109) and carried out according to guidelines for animal experimentation of Hokkaido University, where Mab Institute Inc is located. Animals were housed in a specific pathogen–free facility at the Hokkaido University. Humane euthanasia of mice was performed by cervical dislocation by individuals with a demonstrated high degree of technical proficiency.

### Antibodies and Fab fragment preparation

To generate a monoclonal antibody directed against phosphorylated histone H3 Ser28 (H3S28ph), mice were immunized with a synthetic peptide KQLATKAAR(me3-K)(phospho-S)APATGGVKC coupled to keyhole limpet hemocyanin. After generating hybridomas, clones were screened by ELISA using peptides listed in [22]. A clone CMA315 reacted with H3S28ph regardless of the methylation levels on H3 Lys 27 (H3K27). CMA315 were isotyped as IgG2a-κ using a kit (Serotec; MMT-1). The specific binding of IgG and Fab fragments with cellular H3S28ph was analyzed by western blotting and immunofluorescence, as described previously [22]. For antibody purification, cells were grown in CD Hybridoma medium (Life Technologies) and the supernatant (250 ml) was passed through a HiTrap Protein A HP Sepharose column (1 ml; GE Healthcare). After eluting with 0.1 M glycine-HCl (pH 2.8), the buffer was immediately neutralized with 1.5 M Tris-HCl (pH 8.8). The purified IgG was dialyzed against PBS and concentrated up to 2.5 mg/ml using an Amicon Ultra 15 (50 k-cut off; Millipore). Fab fragments were prepared using a kit (Pierce Fab Preparation Kit 44985; Thermo Scientific) according to the manufacturer's instructions, the buffer was then replaced to PBS using a PD10 desalting column (Bio-Rad) and the samples were concentrated using an Ultrafree 0.5 filter (10 k-cut off; Millipore). H3 Lys9 acetylation (H3K9ac)-specific antibody and Fab fragment (CMA310 and Fab310, respectively) were described [15]. The binding affinity of Fab fragments to the epitope was measured by surface plasmon resonance (SPR) using a BIACORE ×100 (GE Healthcare Biosciences), as described previously [15].

### Dye labeling

Purified Fab was conjugated with the fluorescent dyes listed in Table 1. Dyes were dissolved into dimethyl sulfoxide (DMSO; Wako) at 0.1 mg/ml and stored at −20°C. Fab fragments (100 µg) were diluted into 0.1 M NaHCO_3_ (pH 8.3) in 100 µl. After addition of a dye solution (0.2–4.0 µl), the mixture was incubated for 1 h at room temperature using a rotator. The sample was passed through a desalting column (GE Healthcare) to remove unconjugated dye molecules, and dye-conjugated Fab fragments were concentrated using an Ultrafree 0.5 filter (10 k-cut off; Millipore).

**Table 1:** Property of dyes.

Color	Dye	Molecular mass^a^	Ex. max	Em. max	Net charge	Correction factor	Extinction coefficient	Spacer length	Source
Green	Alexa Fluor 488	825(643)	495	519	−2	0.11	71000	0	Life Technologies; A30052
	ATTO 488	981	501	523	−1	0.1	90000	5	ATTO-TEC; AD488-31
	Chromeo 488	615	488	517	0	0.16	73000	1	Active Motif; 15511
	Dylight 488	1011	493	518	NA^b^	0.147	70000	NA	Thermo; 046403
	HiLyte 488	698.6	502	527	NA	0.27^c^	70000	NA	AnaSpec; 81161-1
Red	Alexa Fluor 555	∼1250	555	565	NA	0.08	150000	NA	Life Technologies; A37571
	ATTO 550	694	554	576	NA	0.12	90000	NA	ATTO-TEC; AD555-31
	BODIPY TMR-X	608.5	535	574	0	0.05	50000	10	Life Technologies; D-6117
	CF 555	∼900	555	565	−3	0.08	150000	6	Biotium; 92130
	Chromeo 546	703.8	545	561	0	0.093	96800	8	Active Motif; 15211
	Cy3	766	550	570	−1	0.08	150000	5	GE Healthcare; PA23001
	Dy547	NA	557	574	−1	0.106	150000	3	DYOMICS; 547-01
	Dy548	NA	558	572	−2	0.084	150000	3	DYOMICS; 548-01
	Dy549	NA	560	575	−3	0.08	150000	3	DYOMICS; 549-01
	HiLyte 555	1067.36	552	569	NA	0.1	150000	NA	Dojindo; LK14
	Dylight 550	1040	562	576	NA	0.081	150000	NA	Thermo; 62263
	Rhodamine	528	552	575	0	0.34	80000	1	Thermo; 46406
Far Red	Alexa Fluor 647	∼1300	650	665	NA	0.03	239000	NA	Life Technologies; A37573
	ATTO 655	887	663	684	NA	0.08	125000	NA	ATTO-TEC; AD655-31
	CF633	(820)	630	650	−1	0.48	100000	7	Biotium; 92133
	CF640R	∼1034 (832)	642	662	−1	0.05	105000	6	Biotium; 92108
	Chromeo642	701.8	642	660	0	0.027	180000	6	Active Motif; 15311
	Cy5	792	650	670	−1	0.05	250000	5	GE Healthcare; PA25001
	Dylight 650	1066	652	672	NA	0.037	250000	NA	Thermo; 62266

a The list here contains NHS ester weight, ():fluorophore only.

b Not available.

c As it was not supplied from the manufacturer, we estimated the value by measuring the ratio of A_280_∶A_488_.

The concentration of Fab fragments and dye∶protein ratios were calculated from the absorbance at 280 and 488, 543, or 647 nm, measured using a spectrophotometer (Nanodrop), using the following formula:
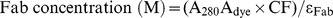


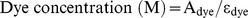


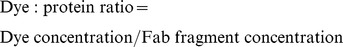
where A_280_ is the measured absorbance of dye-conjugated Fab fragments for a 1 cm path length at 280 nm, A_dye_ is the maximal absorbance of the dye used for conjugation (i.e. the absorbance at either 488 nm, 543 nm, or 647 nm), CF is the correction factor (i.e., the ratio of the absorbance at 280 nm for the dye alone to A_dye_), ε_dye_ is the extinction coefficient of each dye at the excitation wavelength (Table 1), and ε_Fab_ is the extinction coefficient of Fab fragments at 280 nm (70,000 M^−1^cm^−1^; one-third of IgG).

### Live cell imaging and immunofluorescence

HeLa cells were grown in Dulbecco's modified Eagle's medium, high-glucose (Nacalai Tesque) supplemented with L-Glutamine–Penicillin–Streptomycin solution (Sigma) and 10% fetal calf serum (JRH). For live cell imaging, cells were plated on a glass-bottom dish (Mat-Tek) and loaded with fluorescent Fab fragments using glass beads (1 mg/ml Fab; 2 µl) [15], [23] or by microinjection using Femtotip (Eppendorf; 0.2 mg/ml). The dish was placed on an inverted microscope (Ti-E; Nikon) with a PlanApo VC 100× (NA = 1.4) oil-immersion objective lens, featuring a culture system (Tokai Hit) at 37°C under 5% CO_2_. Fluorescent images were captured under the operation of NIS Elements ver. 3.0 (Nikon) using an EM-CCD (iXon+; Andor; Conversion gain 5.1×; Normal mode). A filter set LF488-A (Semrock; Ex FF02-482/18; DM Di02-R488; Em FF01-525-45), TRITC-A (Semrock; Ex FF01-543/22; DM FF562-Di03; Em FF01-593/40), or Cy5-4040A (Semrock; Ex FF02-628/40; DM FF660-Di02; Em FF01-692/40) was used for green, red, or far-red fluorescent dyes, with a 75 W Xenon lamp and a 440 nm long-pass filter. When necessary, phase-contrast images were also collected using an external phase ring. For the photostability assay, cells were exposed to excitation light without neutral-density filters. Excitation light powers out of a PlanApo VC 100× objective lens, measured using a power meter (ADCMT, 8230E), were 630, 430 and 540 mW for LF488-A, TRITC-A, and Cy5-4040A, respectively. Image analyses were performed using Mathematica software (ver. 9; Wolfram Research).

To evaluate dye-conjugated Fab315, 6–7 cells in metaphase were analyzed. After the acquisition of 6 z-stack images with 1 µm intervals, maximum intensity projection images were created and the intensity of background (outside the cells) was subtracted. To select cytoplasmic and chromosome areas, the images were cropped to contain single cells and normalized to have values between 0 and 1 by setting the maximum intensities to 1 (the minimum was 0 due to the background subtraction). The areas of the cell and chromosomes were selected by binarization using intensity thresholding by visual inspection. As maximum projection images of metaphase cells were used, all chromosomes were converged in a single area. After defining these areas, the net intensities in the chromosome and cytoplasmic areas were measured and the chromosome∶cytoplasm ratio was calculated. To detect cytoplasmic aggregation spots on maximum projection images, the position and edges of spots were detected using a spatial bandpass filter with spatial wavelength cutoff values chosen to highlight objects within a designated size range (using custom Mathematica code, available upon request). The areas of spots were typically 6–125 pixels (0.15–3.2 µm^2^). The spot number and area size appeared to be correlated; when there were more spots, the spot size was typically bigger.

To evaluate dye-conjugated Fab310, 6–7 interphase cells were analyzed. The nuclear area was selected as above (background subtraction, normalization, and binarization), and the surrounding area (pixels within a 4.8 µm width donut surrounding the nucleus) was used to define the cytoplasm (using custom Mathematica code, available upon request). The fluorescence intensities in the nucleus and cytoplasm were measured, and the nucleus∶cytoplasm ratio was calculated. For the photostability assay, the fluorescence intensity in individual nuclei was measured over time. After background subtraction, the net intensities were plotted and fit to a single exponential decay curve having the form *I(t)* = e^-k*t*^, where *I(t)* is the intensity at time (*t*) and k is the bleach coefficient. If the curve could not be fit by a single exponential (Chromeo488 and Dylight488), a double exponential was used: *I(t)* = P1*e^-k*1t*^+P2*e^-k*2t*^, where k*1* and k*2* are the bleach coefficients and *P1* and *P2* are the fractions of the two bleached populations. The half-time (*t_1/2_*) was calculated using the following formula: *t_1/2_* = ln2/k (single exponential) or *t/_1/2_* = P1*ln2/k*1*+P2*ln2/k*2* (double exponential).

For immunofluorescence using fixed samples, HeLa cells grown on coverslips (No. 1-S, Matsunami) were fixed with 1% formaldehyde (Electron Microscopy Sciences) in 250 mM Hepes-NaOH (pH 7.4; Wako) containing 0.1% Triton X-100 (Nacalai Tesque) for 10 min at room temperature, permeabilzed with 1% Triton X-100 in phosphate-buffered saline (PBS; Wako) for 20 min, and washed three times with PBS. After blocking with Blocking One-P (Nacalai Tesque) for 15 min, cells were incubated in 1 µg/ml dye-labeled Fab fragments in PBS containing 10% Blocking One-P for 2 h. Next cells were washed three times with PBS containing 0.05% Tween 20 (Wako) over 30 min and DNA was counterstained with 100 ng/ml 4′,6-diamidino-2-phenylindole (DAPI; Nacalai Tesque) in PBS for 20 min. Following this, cells were washed again with PBS and coverslips were mounted using Prolong-Gold (Life Technologies). Fluorescence images were collected using a fluorescence microscope (Ti-E; Nikon), as described above.

## Results

### Effect of fluorescence∶protein ratio on epitope binding of H3S28ph-specific Fab fragment (Fab315) in living cells

**Figure 1:**
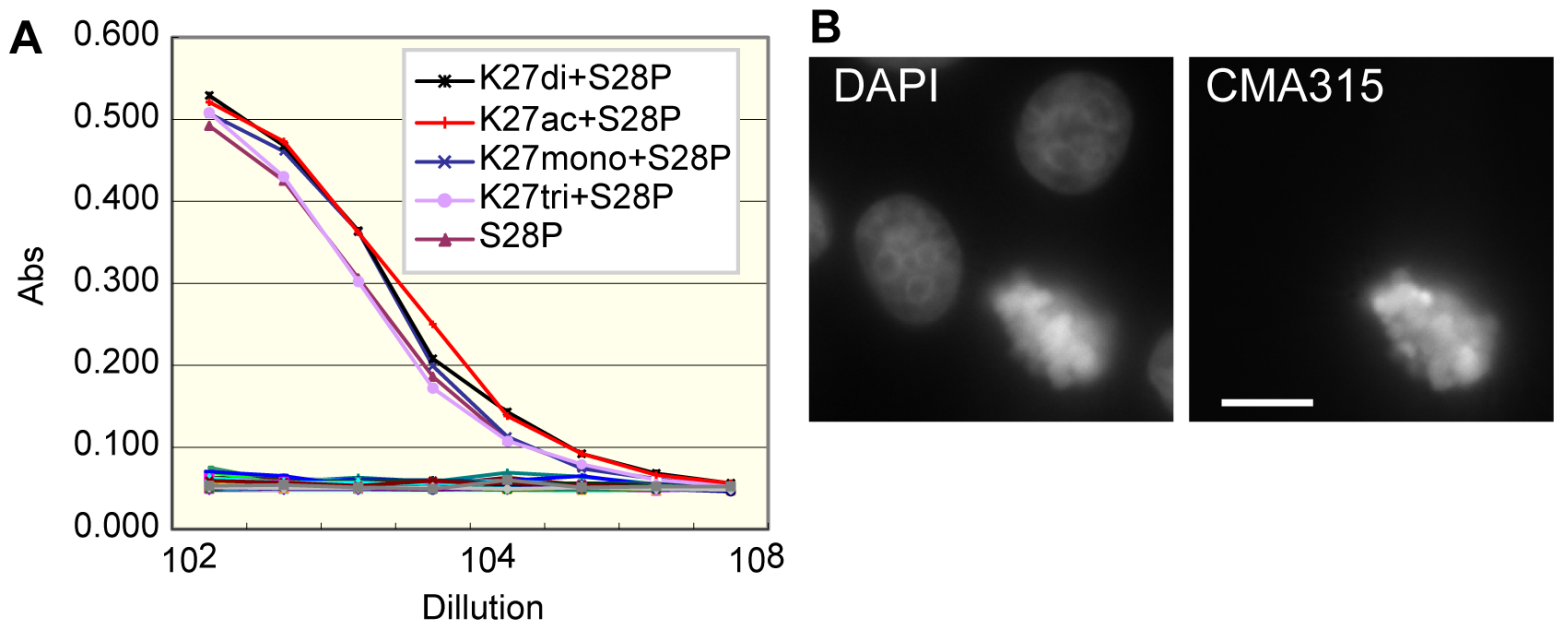
CMA315 specifically reacts with phoshorylated histone H3S28 (H3S28ph). (**A**) ELISA. Microtiter plates coated with the peptides listed in [22] were incubated with 3-fold dilutions of the antibody (CMA315), starting from a 1∶100 dilution of a hybridoma culture supernatant. After incubation with peroxidase-conjugated secondary antibody and washing, the colorimetric signal of tetramethylbenzidine was detected by measuring the absorbance at 405 nm (Abs) using a plate reader. CMA315 exclusively bound to peptides containing phosphorylated S28 (inset). (**B**) Immunostaining. HeLa cells were fixed and stained with CMA315 and then Alexa Fluor 488-conjugated anti-mouse IgG. Condensed chromosomes in prophase and metaphase cells were stained. Bar 10 µm.

To assess the suitability of different fluorescent dyes for live cell imaging using dye-conjugated proteins, we used a new monoclonal antibody directed against histone H3 phosphorylated at Ser28 (H3S28ph). This modification occurs almost exclusively on condensed chromosomes in mitotic cells, so the binding properties of a specific antibody are easily evaluated by its localization. A monoclonal antibody CMA315 reacted specifically with peptides harboring H3S28ph by ELISA, regardless of the methylation status of neighboring H3K27 (Fig. 1A). As expected, mitotic chromosomes were highlighted by immunofluorescence (Fig. 1B). As these results indicated CMA315 specifically reacts with cellular H3S28ph, we prepared Fab fragments (termed Fab315) for live cell imaging.

**Figure 2:**
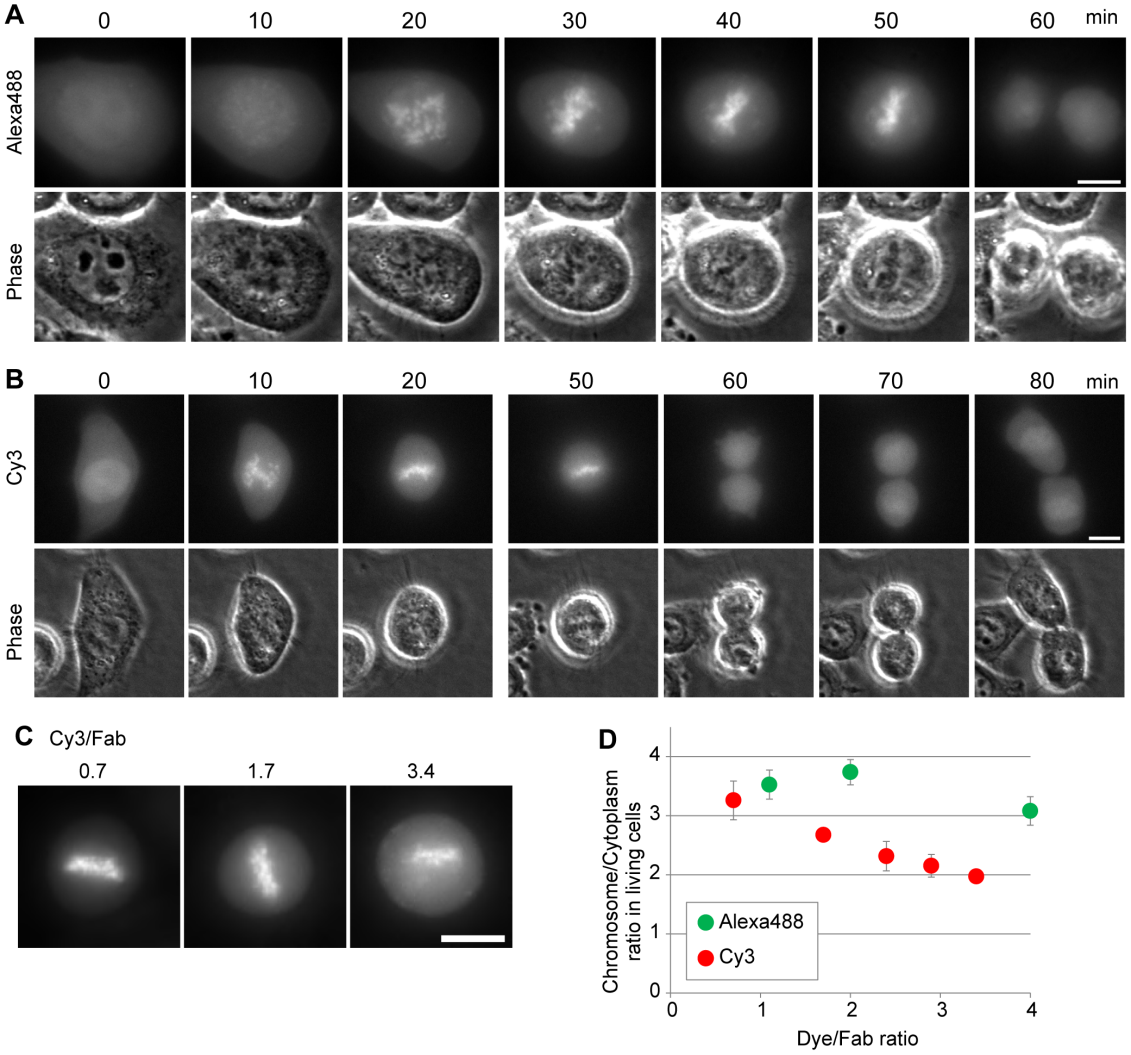
Concentration of Fab315 on mitotic chromosomes in living cells and the effect of dye∶Fab fragment ratio. Fab315 was conjugated with Alexa488 or Cy3 with different dye∶Fab ratios, and loaded into HeLa cells. (**A** and **B**) Time-lapse imaging of cells loaded with Fab315 conjugated with Alexa488 (dye∶Fab ratio 2.0; **A**) and with Cy3 (dye∶Fab ratio 1.7; **B**). Fluorescence and phase-contrast images are shown. In either case, dye-labeled Fab315 is concentrated on mitotic chromosomes, where H3S28 is phosphorylated. (**C**) Representative images of mitotic cells harboring Fab315 conjugated with Cy3 at different dye∶Fab ratios. (**D**) Chromosome∶cytoplasm ratio of Fab315 conjugated with Alexa488 or Cy3 at different dye∶Fab ratios (average with s.d.; n = 6). Bars 10 µm.

We first conjugated Fab315 with Alexa Fluor 488 (Alexa488) and Cy3. When loaded into HeLa cells, labeled Fab315 were evenly distributed throughout interphase cells and became concentrated on condensed chromosomes, in which H3S28 is phosphorylated, in mitotic cells (e.g., Fig. 2A and 2B). After chromosome separation, Fab315 dissociated from decondensing chromosomes, consistent with the dynamic binding of Fab fragments to their targets in living cells [14], [15].

To investigate the effect of the number of conjugated dye per Fab molecule, we conjugated Fab315 with different concentrations of amine-reactive Alexa488 or Cy3. Resulting dye-labeled Fab315 yielded dye∶protein ratios of 1.1, 2.0, and 4.0 for Alexa488, and 0.7, 1.7, 2.4, 2.9 and 3.4 for Cy3. We then compared the enrichments of differentially labeled Fab315 on mitotic chromosomes by measuring the chromosome∶cytoplasm intensity ratio (Fig. 2C and 2D). For Alexa488-labeled Fab315, the chromosome∶cytoplasm intensity ratios (i.e., Fab315 enrichment on chromosomes) were similar at dye∶protein ratios of 1.1 and 2.0, and slightly decreased at 4.0 (Fig. 2D). For Cy3-labeled Fab315, the chromosome∶cytoplasm intensity ratio appeared to anti-correlate with the dye∶protein ratio (Fig. 2C and 2D), suggesting that Fab315 binding affinity to the epitope was partially blocked by multiple conjugated Cy3 molecules, leading to an increased unbound fraction.

The different effect between dyes is not likely due to the conjugation sites as both dyes were amine-reactive derivatives that crosslink to lysine residues. Rather, the chemical properties of the dyes and linkers are likely to affect the function of Fab fragments. Thus, to fairly compare the effects of different dyes on Fab function in living cells, we adjusted the labeling conditions for each fluorophore to yield the optimal dye∶protein ratio between ∼1–2.

### Immunofluorescence and live cell localization of H3S28ph-specific Fab315 labeled with various dyes

**Figure 3:**
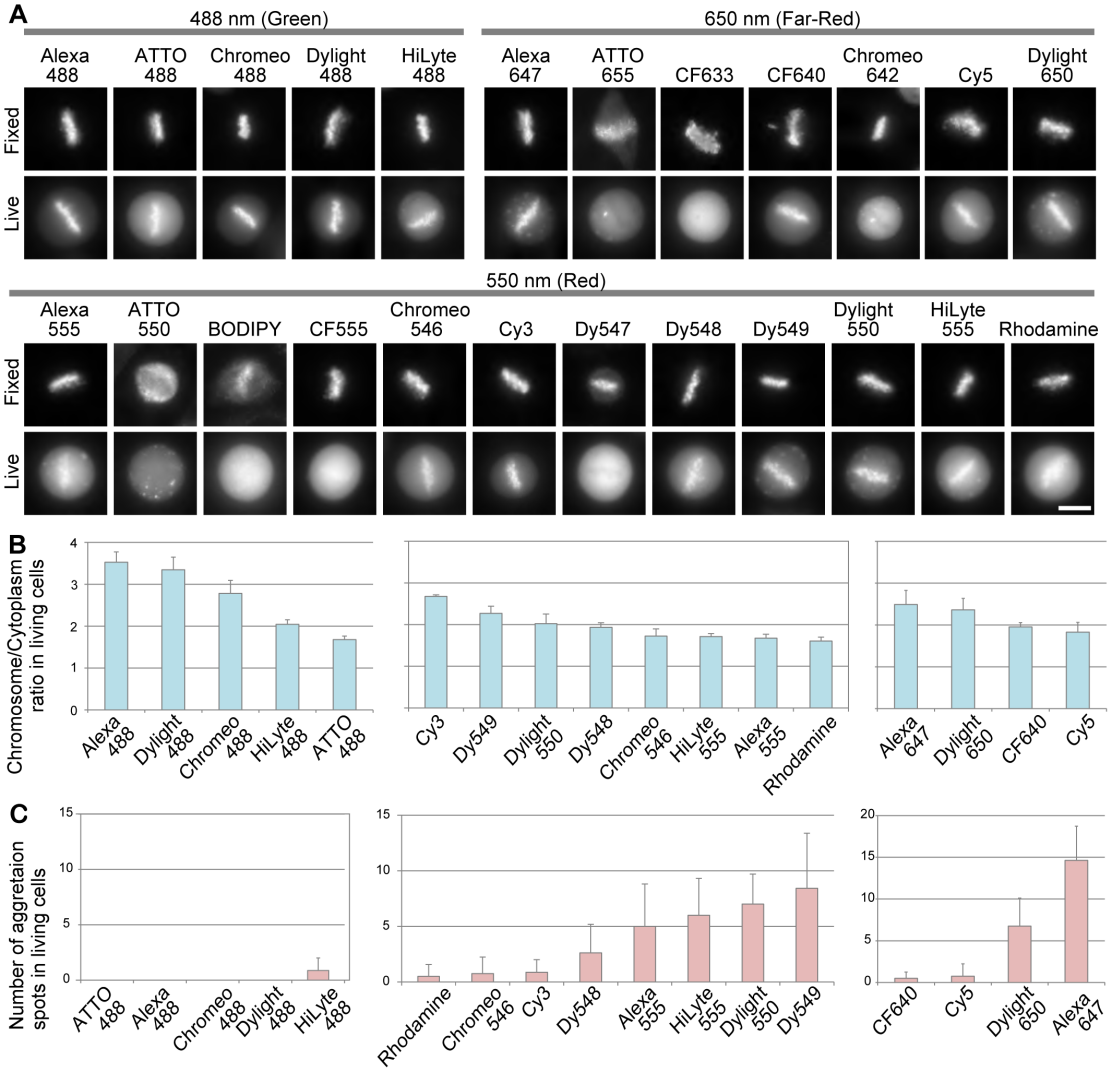
Distribution of Fab315 conjugated with different fluorescent dyes in fixed and living cells. (**A**) Representative images of mitotic cells stained or loaded with dye-labeled Fab315. HeLa cells were fixed, permeabilized, and stained (top rows; Fixed) or loaded (bottom rows; Living) with Fab315 conjugated with different dyes. Fluorescence images of metaphase cells where chromosomes were aligned in the middle were collected 4–6 h after loading. Most dye-labeled Fab315 highlighted chromosomes in fixed cells with low background. In living cells, some were concentrated on chromosomes and others were not. Bar 10 µm. (**B**) Enrichment of dye-labeled Fab315 in mitotic chromosomes in living cells. Using images like those shown in (**A**), fluorescence signal intensities in chromosome and cytoplasm areas were measured, and the chromosome∶cytoplasm intensity ratios were plotted (averages with s.d.; n = 6–7). The dyes showing higher chromosome∶cytoplasm ratios are suitable for live imaging. (**C**) The number of cytoplasmic spots. z-stack fluorescence images (6 stacks; 1 µm intervals) of cells in metaphase were collected and maximum intensity projection images were analyzed. Cytoplasmic spots were detected with a help of edge highlighting, and the numbers were counted and plotted (averages with s.d.; n = 6–7). The number of spots may be underestimated because projected images were analyzed, but this information is still useful for the comparison among different dyes. The dyes with lower number of spots are suitable for live imaging.

We conjugated Fab315 with various commercially available fluorescent dyes, including 5 green, 12 red, and 7 far-red amine-reactive dyes, whose excitation wavelength are ∼488 nm, ∼555 nm, and ∼650 nm, respectively (Table 1). The dye∶protein ratio and assay results are summarized in Table 2. Firstly, to confirm the epitope binding ability of these dye-labeled Fab315, immunofluorescence was performed using fixed HeLa cells. As expected, most of them were concentrated on mitotic chromosomes (Fig. 3A, Fixed). However, Fab315 labeled with ATTO550 failed to stain mitotic chromosomes, and some others (labeled with ATTO655, BODIPY-TMR-X, and Dy547) exhibited less specific staining and higher background (Fig. 3A, Fixed), indicating that these dyes severely interfered with Fab315 function. Secondly, to see whether labeled Fab315 can bind to mitotic chromosomes in living cells, they were loaded into HeLa cells and their distribution was analyzed 4–6 h later (Fig. 3A, Live). Labeled Fab 315 that did not stain mitotic chromosomes by immunofluorescence with fixed cells also failed to be concentrated on chromosomes in living cells. In addition, Fab315 labeled with CF555, CF633 and Chromeo642 did not highlight mitotic chromosomes. Other labeled Fab315 were concentrated on mitotic chromosomes, but the contrast to cytoplasmic signals varied. Some labeled Fab315 also exhibited unwanted spotty signals in the cytoplasm, which can be aggregation or accumulation in cytoplasmic vesicles. The formation of cytoplasmic spots is not likely due to transient stress from protein loading by glass beads, since spots become apparent only several hours after loading and also appeared when a milder microinjection method was used (Fig. S1). We then measured the enrichment of signals on chromosomes over the cytoplasm (Fig. 3B), and the number of cytoplasmic spots (Fig. 3C).

**Table 2:** Summary of dye-conjugated Fab fragment.

Color	Dye	Fab315				Fab310	
		Dye∶Fab ratio	Chromosomal localization^a^		Cytoplasmic foci^b^	Dye∶Fab ratio	Nuclear localization^c^
			Fixed	Living			
Green	AlexaFluor488	1.1	++	++	none	1.6	++
	ATTO488	1.3	++	+	none	1.9	++
	Chromoe488	1.1	++	++	none	0.9	+
	Dylight488	2.0	++	++	none	1.6	++
	HiLyte488	1.0	++	++	few	NT^e^	NT
Red	AlexaFluor555	2.0	++	+	several	NT	NT
	ATTO550	2.0	−	−	ND^d^	NT	NT
	BODIPY-TMR-X	1.8	+	−	ND	NT	NT
	CF555	1.3	++	−	ND	NT	NT
	Chromeo546	0.9	++	+	few	1.9	+
	Cy3	1.7	++	++	few	0.9	++
	Dy547	1.8	++	−	ND	NT	NT
	Dy548	0.9	++	++	several	1.6	++
	Dy549	1.7	++	++	many	NT	NT
	Dylight550	1.6	++	++	many	NT	NT
	HiLyte555	1.0	++	+	many	NT	NT
	Rhodamine	1.8	++	+	few	1.7	++
Far Red	AlexaFluor647	1.3	++	++	many	NT	NT
	ATTO655	2.0	+	−	ND	NT	NT
	CF633	1.4	++	−	ND	NT	NT
	CF640	0.9	++	++	few	1.2	+
	Chromoe642	1.2	++	−	ND	NT	NT
	Cy5	1.8	++	++	few	1.2	++
	Dylight650	2.0	++	++	many	1.7	+

a Enrichment of dye-labeled Fab315 on mitotic chromosomes in fixed and living cells. The degree of enrichment is indicated by ++ (strong), + (medium), or − (none). For living cells, ++ and + indicate >1.8- and <1.8-fold enrichment, respectively.

b Degree of cytoplasmic foci formation is indicated as none (no focus), few (<3), several (3∼8), or many (>8).

c Enrichment of dye-labeled Fab310 in the nucleus in living cells. The degree of enrichment is indicated by ++ (>5-fold enrichment) or + (<5-fold).

d Not determined.

e Not tested.

The green fluorescent dyes showed relatively high chromosome∶cytoplasm intensity ratios compared to red and far-red dyes (Fig. 3B) and cytoplasmic spots were barely observed (Fig. 3C). In particular, Alexa488, Dylight488, and Chromeo488 showed superior performance with high contrast and without spots. Among red fluorescent dyes, Cy3, Dy549, Dylight550, and Dy548 showed higher contrast (Fig. 3B, middle). However, Dy549 and Dylight550 were associated with a large numbers of cytoplasmic spots (Fig. 3C, middle), indicating that these dyes are not so suitable for live cell imaging. Therefore Cy3 might be the first choice among red fluorescent dyes. Among far-red fluorescent dyes, Alexa647 and Dylight650 showed the highest contrast, but were also prone to accumulation into many cytoplasmic spots. CF640 and Cy5, which showed only a small number of spots, appeared rather better choices.

### In vitro binding affinity of dye-labeled Fab315

**Figure 4:**
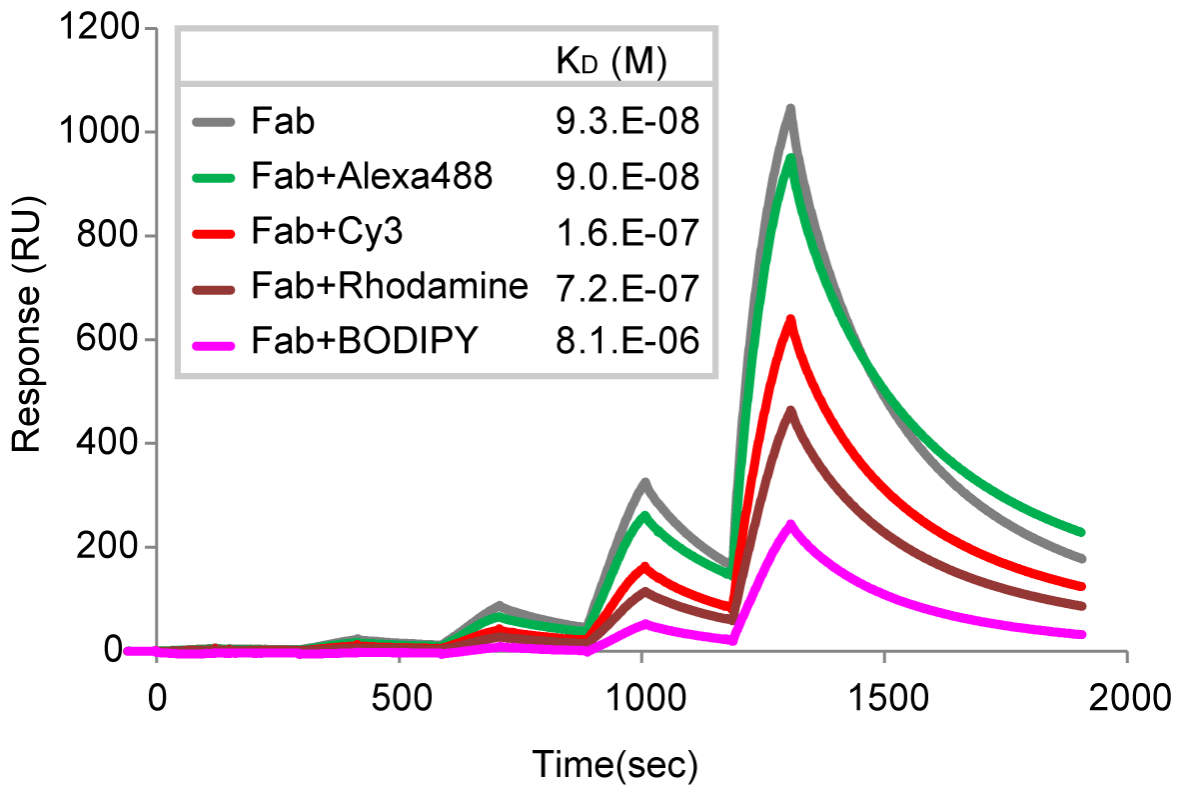
Surface plasmon resonance (SPR) measurements of Fab315 binding affinity. Binding profiles of Fab315 and its dye-conjugated forms to the epitope peptide are shown with the dissociation constants (K_D_). Fab fragments were injected 5 times at different concentrations (low to high). K_D_ was obtained by built-in software for curve fitting.

When conjugated to Fab315, dyes could directly affect the Fab property by interacting with epitope binding regions and/or by steric hindrance. To investigate the effect of dye-conjugation on the epitope binding affinity of Fab315 in vitro, surface plasmon resonance (SPR) measurements were performed using a Biacore system. As shown in Fig. 4, unconjugated and Alexa488-labeled Fab315 bind most strongly to the target phospho-peptide with the dissociation coefficient (K_D_) ∼9×10^−8^ M. The binding affinity of Cy3-labled Fab315 was slightly lower (K_D_ 1.6×10^−7^ M), and further lowered affinities were observed for tetramethylrhodamine (Rhodamine)- and BODIPY-TMR-X-labeled Fab315 (K_D_ 7.2×10^−7^ and 8.1×10^−6^ M, respectively). These results indicate that dye-conjugation as such can interfere with the binding affinity of Fab315, depending on the chemical property of individual dyes. Therefore, the different affinity in dye-labeled Fab fragments could, at least partly, explain their different performance in living cells.

### Effects of different fluorescent dyes on H3K9ac-specific Fab fragment (Fab310) in living cells

**Figure 5:**
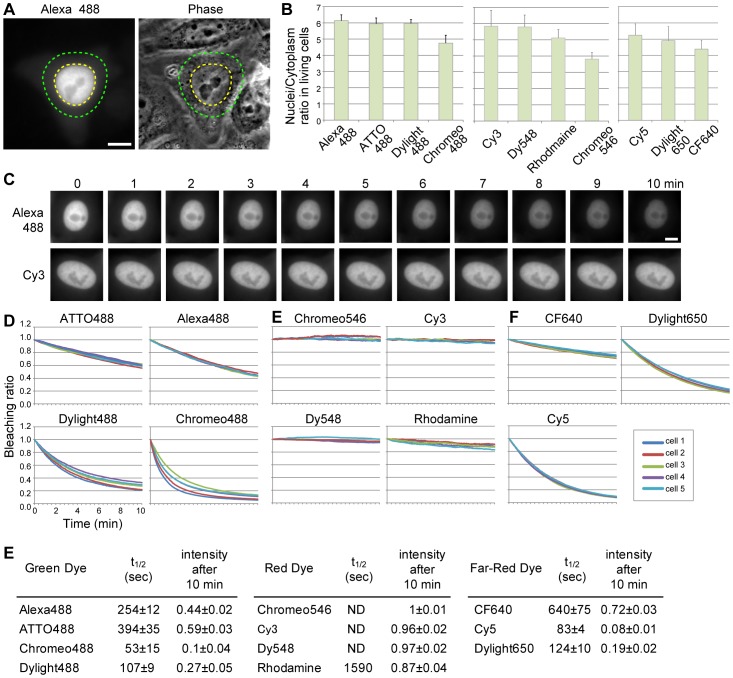
Distribution and photostability of Fab310 conjugated with different fluorescent dyes in living cells. (**A** and **B**) Enrichment of dye-labeled Fab310 in the nucleus in living cells. HeLa cells were loaded with dye-labeled Fab310 and single focal planes were collected 4–6 h after loading. Fluorescence and phase-contrast images of a typical cell loaded with Alexa488-labeled Fab310 are shown in (**A**). Bar 10 µm. The enrichment of Fab310 in the nucleus was quantified by measuring the fluorescence intensities in the nucleus (yellow dotted area) and surrounding cytoplasm (4.8 µm-width area outside the nucleus, between yellow and green dots). The nucleus∶cytoplasm ratios of dye-labeled Fab310 (averages with s.d.; n = 6–7) are plotted in (**B**). The dyes showing higher nucleus∶cytoplasm ratios are suitable for live imaging. (**C**–**E**) Photostability of dyes conjugated with Fab310. HeLa cells loaded with dye-labeled Fab310 were continually exposed to excitation light for 10 min, during which images were captured every 2 sec. (**C**) Example image series for Alexa488- and Cy3-labled Fab310. The intensity of Alexa488 became apparently dimmer while that of Cy3 remained constant. Bar 10 µm. (**D**) Changes in fluorescence intensity by continuous excitation. Fluorescence intensities in nuclei were measured and plotted as relative values to the first image. Five different cells were analyzed for each dye-labeled Fab310. (**E**) Summary of photostability. The curves shown in (**D**) were fit to exponential decay curves. The bleach half time (t_1/2_) and relative intensity after 10 min exposure are shown with s.d. (n = 5). Due to little bleaching under the condition used in the study, the t_1/2_ could not be determined for Chromeo546, Cy3, and Dy548 (ND; not determined). The dyes showing less bleaching are suitable for live imaging.

To examine whether the different dye properties are also observed when conjugated to other Fab fragments, we labeled Fab310, which is derived from monoclonal antibody (CMA310) directed against histone H3 Lysine 9 acetylation (H3K9ac). Among the fluorescent dyes we tested above, dyes that performed relatively well were used for conjugation with Fab310. When loaded into HeLa cells, all labeled Fab310 were concentrated in the nucleus where the target H3K9ac is present (Fig. 5A), consistent with a previous study using Alexa488-labeled Fab310 [15]. To compare the bound fraction of labeled Fab310, the nucleus∶cytoplasm intensity ratios were measured (Fig. 5B). Again, green fluorescent dyes, except Chromeo488, showed the highest enrichments in nuclei. Among red fluorescent dyes, Cy3 and Dy548 were more concentrated in nuclei than Rhodamine and Chromeo546. Far-red fluorescent dyes showed the lowest enrichments, but among them, Cy5 and Dylight650 were better than CF640. These results were similar to those for Fab315 conjugations.

### Photostability of different dyes in living cells

Finally, we measured the photostability of these dyes conjugated to Fab fragments in living cells (Fig. 5C–D). For these purposes, we chose Fab 310 because of their strong nuclear localization throughout interphase (as opposed to Fab315, which are concentrated on rapidly-moving mitotic chromatin). HeLa cells loaded with dye-labeled Fab310 were continuously exposed to excitation light for 10 min (with 2.0 sec camera capturing time) (Fig. 5C), and fluorescence intensities in the nucleus were measured over time (Fig. 5D). Among green fluorescent dyes, ATTO488 was most photostable and Alexa488 was slightly less stable. In contrast, Dylight488 and Chromeo488 were rapidly photobleached. Red fluorescent dyes were all very stable under the experimental conditions, with a slight bleaching of Rhodamine. Among Far-red dyes, CF640 was more photostable than the others (i.e., Dylight649 and Cy5). Thus, the photostability in living cells can vary among different dyes, and it would be better to choose photostable ones when the other properties are compromised.

## Discussion

In this study, we compared the suitability of a set of commercial fluorescent dyes for live cell imaging when conjugated to Fab fragments. Among the dyes with different excitation/emission wavelengths, green fluorescent dyes (with excitation at ∼488 nm) minimally affected the Fab binding affinity compared to red and far-red fluorescent dyes (with excitation at ∼550 and ∼650 nm, respectively), probably due to their smaller fluorophore structure. Indeed, Fab315 conjugated with any green dyes examined were concentrated into mitotic chromosomes, whereas 4 out of 12 red and 3 out of 7 far-red fluorescent dyes did not show the specific localization. As the chemical structures of most dyes are not disclosed, it is difficult to find out the chemical basis accounting for the properties like cytoplasmic foci formation. Nevertheless, the total molecular size, net charge or spacer length as such does not seem to be a single critical factor (Table 1). For example, the conjugation of Fab315 with Rhodamine and BODIPY resulted in lower affinity than that with Cy3, although Cy3 is bigger in size. The overall structure and specific chemical group may both affect the Fab affinity, since Rhodamine shares the same xanthene backbone as Alexa488 that showed minimal interference. Thus, it is difficult to speculate the inhibitory mechanism by individual dyes. Recent single molecule analysis using anti-EGFR affibody has shown that hydrophobicity can cause non-specific binding on plasma membrane [21].

Among all dyes examined, Alexa488 was the best for conjugation to Fab fragments as determined by three criteria. First, the increased dye∶protein ratio on Fab315 did not significantly alter the chromosome∶cytoplasm intensity ratio in living cells. Second, the enrichment in mitotic chromosomes (Fab315) and the nucleus (Fab310) were the highest. Third, the binding affinity to the epitope peptide was not changed in Alexa488-labeled Fab315 compared to unlabeled Fab315 by an in vitro SPR assay. Dylight488 exhibits similar performance to Alexa488 in living cells; however, it is more susceptible to photobleaching. ATTO488 may be an alternative to Alexa488 because it is more photostable, but its suitability may depend on the specific conjugation partner (as the contrast of Fab315 was low).

Red fluorescent dyes generally showed superb photostability in living cells, even though a direct comparison with other wavelength dyes could not be made because the excitation powers were different (∼630, ∼430, and ∼540 mW excitation for green, red, and far-red dyes, respectively). Another study based on single molecule imaging showed that some red fluorescent dyes, including Cy3 and Alexa555, were more photostable than Alexa488 when conjugated to a protein on cell surface [21]. Among the red dyes, Cy3 appears to be most suitable for live intracellular fluorescence imaging, since the highest contrast was achieved for both Fab315 and Fab310, and cytoplasmic spots were relatively small in number. Unlike Alexa488, however, the affinity of Cy3-labeled Fab was reduced and the dye∶protein ratio was more critical. Chromeo546 showed little cytoplasmic spots, but the enrichments in chromosomes (Fab315) and the nucleus (Fab310) were lower. Although Dy548 may be a better choice than Chromeo546, its supply has been unfortunately discontinued.

Compared to the green and red fluorescent dyes, far-red fluorescent dyes showed lower contrast and a high cytoplasmic spotty background. Among them, Cy5 and CF640 appear to be better, as cytoplasmic spots were less numerous than Alexa647 and Dylight650. Although CF640 was much more resistant to photobleaching than Cy5, the contrast of Fab310 was better when conjugated with Cy5.

So far, the suitability of each dye for live imaging is not predictable and needs to be empirically assessed. Our systematic analysis presented here will be useful for the choice of the most suitable dyes for intracellular fluorescence live imaging application.

## Supporting Information

Figure S1 **Appearance of cytoplasmic spots after microinjection of Fab315.** HeLa cells were microinjected with Cy3- or Alexa555-labeled Fab315, and time-lapse recording was started ∼1 h after injection to capture fluorescence and phase-contrast images every 15 min. (A) Cy3-labeled Fab315. Cytoplasmic spots were barely observed. (B) Alexa555-labeled Fab315. Many cytoplasmic stops appeared (arrows). Bars 10 µm.
(PDF)
