# Supplementary figures and images for: Evaluation of Chemical Fluorescent Dyes as a Protein Conjugation Partner for Live Cell Imaging
Source: PLoS One. 2014 Sep 3;9(9):e106271. doi: 10.1371/journal.pone.0106271 (PMC4153647; doi:10.1371/journal.pone.0106271)

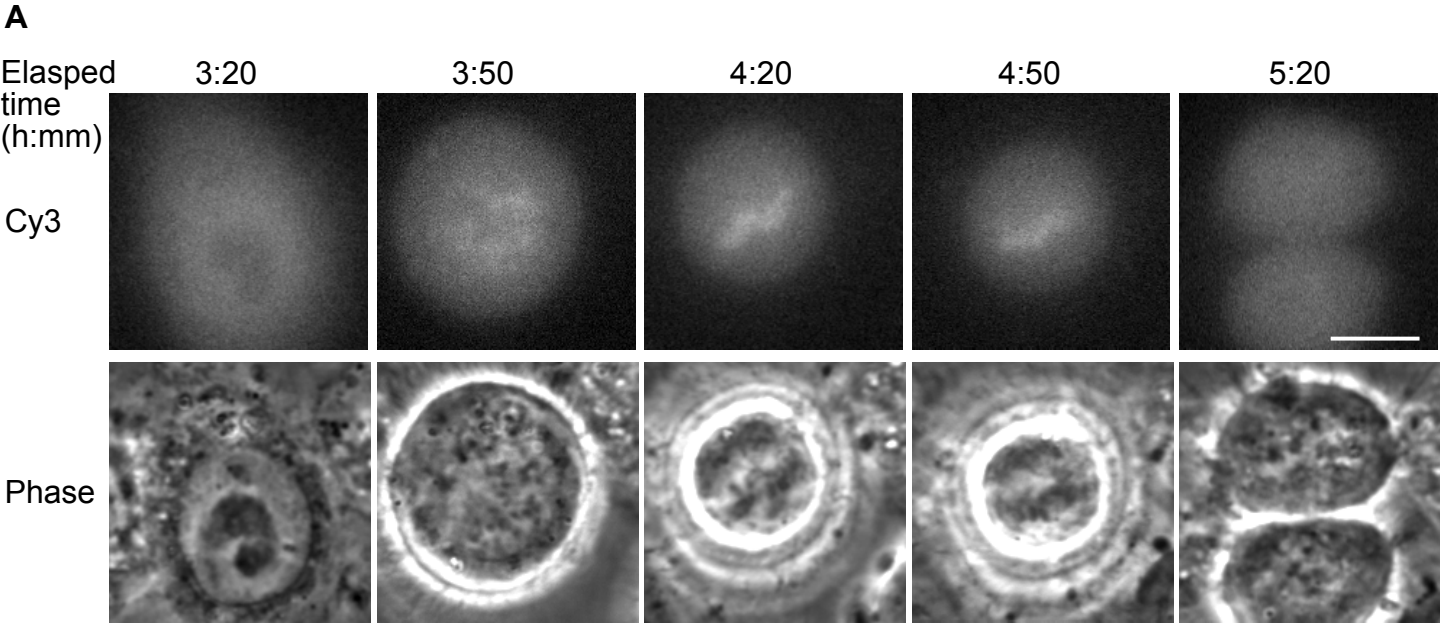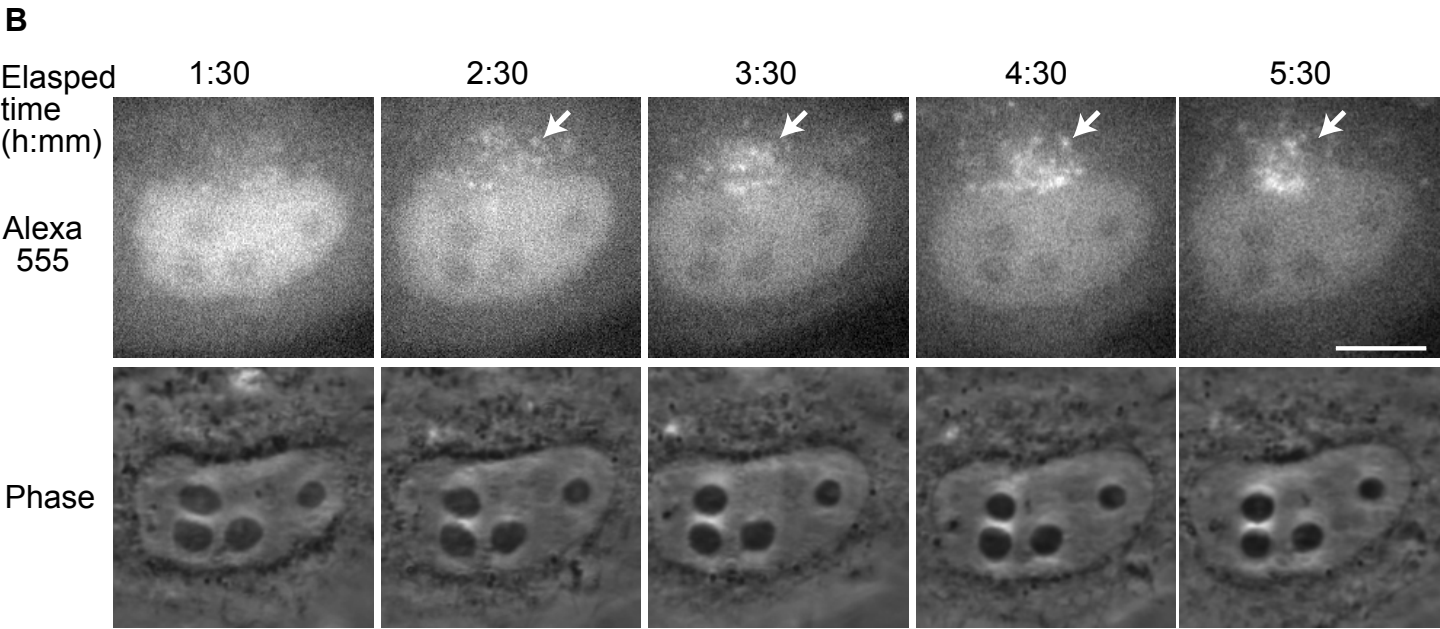

Supplementary Figure 1

Supplement: Figure S1 — Appearance of cytoplasmic spots after microinjection of Fab315. HeLa cells were microinjected with Cy3- or Alexa555-labeled Fab315, and time-lapse recording was started ∼1 h after injection to capture fluorescence and phase-contrast images every 15 min. (A) Cy3-labeled Fab315. Cytoplasmic spots were barely observed. (B) Alexa555-labeled Fab315. Many cytoplasmic stops appeared (arrows). Bars 10 µm. (PDF) [file pone.0106271.s001.pdf]
